# Melatonin from an Antioxidant to a Classic Hormone or a Tissue Factor: Experimental and Clinical Aspects 2019

**DOI:** 10.3390/ijms21103645

**Published:** 2020-05-21

**Authors:** Juan C. Mayo, Rosa M. Sainz

**Affiliations:** 1 Departamento de Morfología y Biología Celular, Redox Biology Group, Instituto Universitario Oncológico del Principado de Asturias (IUOPA), School of Medicine, University of Oviedo. C/ Julián Clavería 6, 33006 Oviedo, Spain; sainzrosa@uniovi.es; 2 Instituto de Investigación Sanitaria del Principado de Asturias (ISPA), Avda. Roma s/n, 33011 Oviedo, Spain

**Keywords:** melatonin, antioxidant, anti-cancer, plant physiology, pineal gland, anti-depressant, neurogenesis, melanoma, preterm infants

## Abstract

During the last 25 years we have accomplished great advances in melatonin research, regarding antioxidant or anti-inflammatory functions, oncostatic actions, glucose metabolism regulation or plant physiology, among others. Of course, we should not forget the classical, circadian-related functions of the indole, which has recently brought up new and important findings. All together these new discoveries will likely lead the way in the next decade in terms of melatonin research. This special issue collects some of these new advances focused on different aspects of the indole

During the past 25 years, research focused on melatonin has experienced a boost in either the number of laboratories involved or the research lines, and, consequently, the number of publications in the field. Thus, while in 1995 there were under 6000 publications, this has risen to more than 26,000 references at present (PubMed database). This increment in such a specific field requires the addition of a Special Issue, in an attempt to collect and funnel the most interesting topics for general public readers, researchers from other areas, or, of course, groups endeavoring towards indole investigation. For this reason, the *International Journal of Molecular Sciences* has promoted the call for publication of articles focused on different aspects of melatonin research, trying to attract the attention of professionals, ranging from basic researchers to clinicians, as the interest in melatonin has grown according to the references published.

During the last two decades, we should highlight three great discoveries in the field that has grabbed the interest of many investigators. The antioxidant properties of melatonin—directly and indirectly [1]—accounts for the cytoprotective effects in so many different tissues and pathologies that would be too long to detail here [2]. Additionally, anti-inflammatory actions have also been linked to these antioxidant properties, and this has been explored thoroughly in several experimental models and patients as well [3,4]. Finally, sometimes related to these antioxidant properties, but which others have explained by different mechanisms, their oncostatic and antitumoral properties also have been reported in a wide variety of tumors [5]. Another significant breakthrough in melatonin research has been the discovery of indole in plants [6,7]. Not only does melatonin exerts essential functions in plant cells, but it does change the evolutionary perspective of indole in living organisms. Finally, we should also mention the important role of melatonin and its receptors in diabetes, insulin secretion, and regulation of blood glucose levels [8,9,10]. This controverted field is currently experiencing a great deal of attention and might bring new discoveries in the next few years. However, all these great advances do not mean that we should set aside more classical circadian-related studies. Instead, the discovery of potential risks for many pathologies associated with light-at-night (LAN) also focus the attention onto the potential role of the drop of night melatonin in nocturnal and shift workers. Altogether, these findings could lead to a second boost of melatonin discoveries in the next decade that would even increase the interest in indole.

In this Special Issue, articles have been selected to bring forth new data about some of the abovementioned topics. It is already well known that melatonin receptors play a role in mediating the cardioprotection of melatonin. Here, Prado and co-workers [11] show how ventricular arrythmias caused by impairment of melatonin secretion, induced by an SCG gangliectomy, are likely mediated by MT1 modulation of the pore-forming subunit (Kir6.1) of the K_ATP_ channels, concluding that the melatonin physiological rhythm exerts cardioprotection.

Two studies focused on the modulatory effects of melatonin on the central nervous system (CNS). In the first report, Ramírez-Rodriguez and co-workers [12] provide insights into the already-reported anti-depressant effect of melatonin. Since neurogenesis at the hippocampal dentate gyrus (DG) help to counteract the effects of antidepressant drugs, they studied how melatonin treatment increased markers of neurogenesis at this region, concomitant with behavioral improvement. On the other hand, Oliveira-Abreu et al. [13] proposed a study using neuron cells from the dorsal root ganglia (DRG). The group has previously classified the complete population of DRG neurons into two types, based on having an inflection (N_inf_), or not (N_0_), on the descending (repolarizing) phase of the action potential (AP). They investigated the melatonin effects on the N_inf_ neuronal population, thus confirming the existence of two different populations of neurons, i.e., excitability melatonin-sensitive and -non-sensitive cells (EMS, EMNS). The study confirms that melatonin modulates several parameters related to the action potential in EMS neurons.

Regarding cancer cells, one of the articles of this Special Issue provides new insights about how melatonin reduces proliferation and wound-healing inhibition in the highly tumorigenic B16F10 melanoma cell line. The report concludes that the profound cytoskeletal reorganization, increase in melanin synthesis, and redox modulation underlie its antiproliferative effects [14].

Hanuszewska and colleagues went deep into the embryological stages in the goose pineal organ, and obtained detailed evidence about the 5-methoxy/hydroxyindoles synthesis [15]. The study showed that melatonin was detectable from ED 18 and, like N-acetyl serotonin, its content increased between ED 22 and ED 28, while diurnal variations appeared from ED 20. In another embryologic study included in this issue, the authors show the impact of melatonin in bovine oocytes and embryos [16]. Physiological serum concentrations of indole increase the cleavage rate, and antagonizes the AKT phosphorylation inhibitor SH6, thus modulating cell death during the development of bovine embryos. Linking melatonin to embryology, Tamura et al. review here the importance of melatonin in assisted reproductive technology [17]. The review includes interesting comments on the trials on humans treated with melatonin, confirming the improvement in assisted reproductive technology, as well as the effect of indole reducing ovarian aging, both with relevant clinical applications in human reproduction.

An interesting collaborative study from several clinical and academic French institutions provides here evidence that the melatonin circulating levels at day of birth are much lower—below detection—in 78% of infants born before 34 GW [18]. This was also confirmed with urine aMT6s concentrations. The report therefore concludes that the clinical use of indole to prevent neurological damage in preterm infants is justified.

Finally, an excellent review by Debnath and colleagues revise the current pieces of evidence of how melatonin modulates the response to stress in plants [19]. The authors highlight the effects of melatonin as a growth regulator, bio-stimulator, and antioxidant. The review also pays attention to how exogenous melatonin enhances the growth, photosynthetic, and antioxidant activities in plants.

All the peer-reviewed articles included in this Special Issue are good examples of the top-quality studies currently performed, and also demonstrates that melatonin research is in a continuing forward dynamic, attracting readers, researchers, and clinicians interested in the different aspects related to indole.
